# Serological profile, seroprevalence and risk factors related to *Lawsonia intracellularis* infection in swine herds from Minas Gerais State, Brazil

**DOI:** 10.1186/s12917-015-0618-z

**Published:** 2015-12-23

**Authors:** Talita Pilar Resende, Carlos Eduardo Real Pereira, Michelle de Paula Gabardo, João Paulo Amaral Haddad, Zélia Inês Portela Lobato, Roberto Maurício Carvalho Guedes

**Affiliations:** Department of Veterinary Clinic and Surgery, Veterinary School Universidade Federal de Minas Gerais, Av. Antônio Carlos, 6627 – Pampulha, Belo Horizonte, MG 31.270-901 Brazil; Department of Preventive Veterinary Medicine, Veterinary School, Universidade Federal de Minas Gerais, Av. Antônio Carlos, 6627 – Pampulha, Belo Horizonte, MG 31.270-901 Brazil

**Keywords:** Proliferative enteropathy, IPMA, Diarrhea, Serology, Antibodies, Epidemiology, Swine

## Abstract

**Background:**

*Lawsonia intracellularis* is the etiologic agent of proliferative enteropathy, which causes diarrhea in several animal species, including swine. Serology can be used both to determine the prevalence of antibodies against a specific pathogen in a herd and to obtain the serological profile, which is used to determine the dynamics of infection in the herd. The objective of this study was to determine the serological profile and seroprevalence of anti-*L. intracellularis* antibodies in swine herds from intensive production regions of Minas Gerais, Brazil, and to identify the risk factors related to the herd-level seropositivity.

**Results:**

A total of 2999 serum samples were collected for this cross–sectional study in the four major regions of intensive swine production in Minas Gerais, Brazil. To obtain better estimates and increase the external validity of the seroprevalence, the sample data were weighted based on the pig population of each herd, the stratum in which the herd was classified and the swine population of the region where each herd was located. A questionnaire was used to identify potential risk factors related to this herd-level seropositivity. The overall weighted prevalence in Minas Gerais was 34.7 % (95 % confidence interval: 32.12 - 37.20 %), and there was no significant difference among the sampled regions, with the seroprevalence rates ranging between 32.06 and 37.66 %. Finishing pigs were the most prevalent among the sampled categories. Among the evaluated risk factors, “cleaning before disinfecting” had a negative impact in the seroprevalence (p < 0.05) and was considered a protective factor.

**Conclusions:**

The anti-*L. intracellularis* antibodies were detected in all of the investigated herds in Minas Gerais, which indicated a wide distribution of the agent in the state. The predominant serological profile was consistent with the dynamics of infection previously observed in pig herds in other countries with similar antimicrobial usage, in which the nursery pigs usually show the lowest seroprevalence and the finishing pigs exhibit the highest. Herds that adopt the practice of “cleaning before disinfection” can decrease their *L. intracellularis* antibody seropositivity.

**Electronic supplementary material:**

The online version of this article (doi:10.1186/s12917-015-0618-z) contains supplementary material, which is available to authorized users.

## Background

Proliferative enteropathy, which is caused by *Lawsonia intracellularis*, is responsible for relevant economic losses in swine production systems due to increased mortality rates, increased drug usage and compromised weight gain [1]. Porcine proliferative enteropathy (PPE) is characterized by enterocyte proliferation and thickening of the intestinal mucosa [2, 3]. It can cause either chronic enteritis, manifested by diarrhea and a reduction in growth rate and hemorrhagic enteritis, which is associated with sudden death in finishing pigs [4], or a subclinical presentation in which the pigs show reduced growth but no clinical signs [5].

Serology is an efficient method for determining swine exposure to a specific agent when sufficient samples are collected [6]. Immunoperoxidase monolayer assay (IPMA) is a serological test with high sensitivity and specificity that can be used to determine the herd serological profile for PPE [7]. Determining the age group in which the peak seroconversion occurs in the herd makes it possible to estimate the time of infection and recommend the use of antibiotics or vaccination, which allows for the development of an active immune response and avoids economic losses from clinical and subclinical diseases [8].

Very few prevalence studies of swine enteric diseases have been conducted in Brazil [9, 10]. Despite the economic impact of PPE [1], risk factor analyses are rare [11, 12].

The purpose of the present study was to determine predominant serological profile to *L. intracellularis* antibodies in intensive pig production regions of Minas Gerais and to identify risk factors related to its infection in pigs and the herd-level seropositivity and the seroprevalence.

## Results

One hundred serum samples were obtained from 30 swine herds; however, one finishing animal sample was lost, resulting in a total of 2999 samples.

### Seroprevalence

**Fig. 1:**
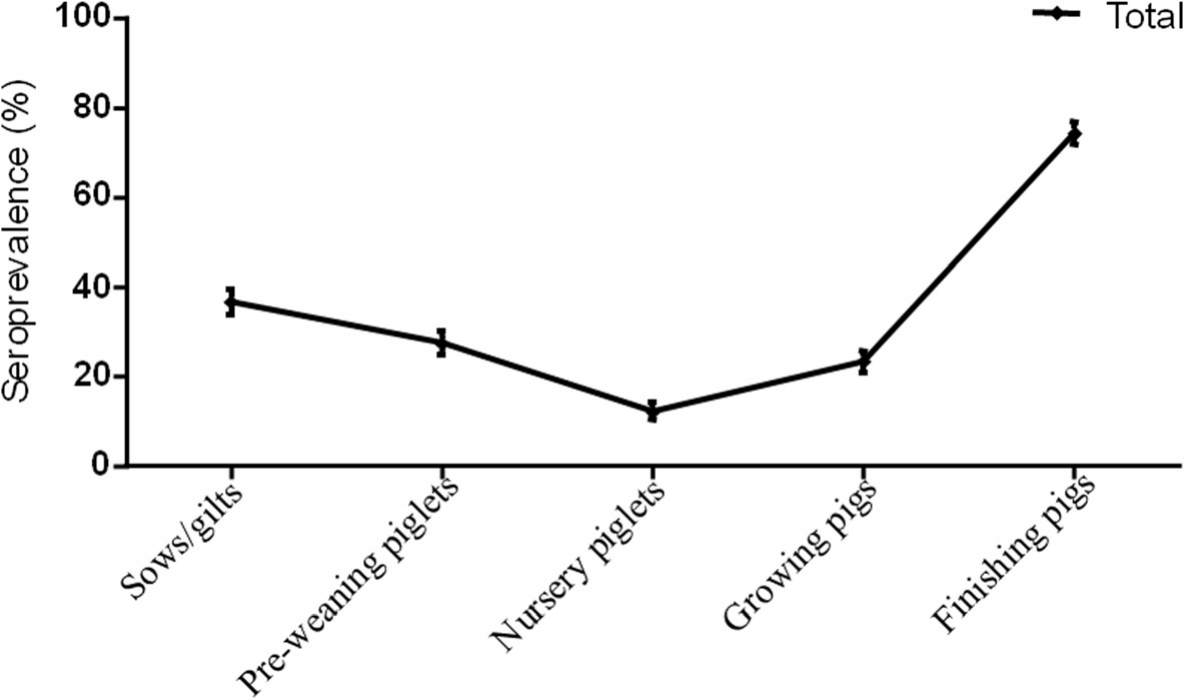
Serological profile for *L. intracellularis* antibodies in swine herds from Minas Gerais state, Brazil. The bars indicate the standard error for the prevalence in each category

All sampled herds had at least one positive serum sample for IgG against *L. intracellularis*. No significant differences were found among the seroprevalences of the analyzed regions (Fig. 1) (Table 1). Regarding the weighted seroprevalence, 34.7 % (95 % Confidence Interval: 32.12 - 37.20 %) of the pigs tested positive for anti-*L. intracellularis* IgG.

**Table 1:** Seroprevalence of anti*-L. intracellularis* antibodies. Seroprevalence results in the four major regions of intensive swine production in Minas Gerais, Brazil, and for the total samples

Region	Number of sampled herds	Seroprevalence	Standard Error	CI 95 %	
				Min.	Max.
MBH	7	37.66 %	2.37 %	33.13 %	42.41 %
ZM	8	32.85 %	1.97 %	29.11 %	36.83 %
SSO	9	32.06 %	2.58 %	27.22 %	37.31 %
TAP	6	35.59 %	2.26 %	31.29 %	40.15 %
Total	30	34.66 %	1.29 %	32.12 %	37.20 %

MBH-Metropolitan Region of Belo Horizonte; ZM-Zona da Mata; SSO-South/South West of Minas Gerais; TAP-Triangulo Mineiro/Alto Paranaíba. CI - confidence interval

### Serological profiles

Serological profiles for each region were drawn according to the seroprevalence found in each stage of the production cycle after weighting the samples (Fig. 1), as well as a general serological profile for Minas Gerais. There were significant differences between phases of the production cycle (Table 2). Generally, finishing pigs had the highest seroprevalence, and nursery pigs had the lowest. Dams had a variable seroprevalence, similar to the results found for piglets and growing pigs.

**Table 2:** Seroprevalence in each category of the swine production cycle

Region	Category	Seroprevalence	Standard error	CI 95 %	
				Min.	Max.
MBH	Sows/gilts ^cA^	40.68 %	5.11 %	31.15 %	50.98 %
	Pre-weaning piglets ^aAB^	40.74 %	5.12 %	31.19 %	51.05 %
	Nursery piglets ^bA^	15.95 %	3.89 %	9.69 %	25.13 %
	Growing pigs ^cA^	11.98 %	3.21 %	6.96 %	19.84 %
	Finishing pigs ^dA^	88.22 %	2.93 %	81.16 %	92.87 %
SSO	Sows/gilts ^cA^	31.34 %	5.38 %	21.83 %	42.72 %
	Pre-weaning piglets ^aC^	12.23 %	3.32 %	7.06 %	20.35 %
	Nursery piglets ^bB^	3.18 %	2.16 %	0.82 %	11.50 %
	Growing pigs ^cA^	36.15 %	5.36 %	26.41 %	47.17 %
	Finishing pigs ^dAB^	65.81 %	4.43 %	56.68 %	73.90 %
ZM	Sows/gilts ^dB^	19.46 %	3.45 %	13.56 %	27.13 %
	Pre-weaning piglets ^aBC^	23.85 %	3.79 %	17.21 %	32.06 %
	Mursery piglets ^bC^	9.91 %	2.62 %	5.82 %	16.38 %
	Growing pigs ^adB^	20.45 %	3.34 %	14.66 %	27.78 %
	Finishingattening pigs ^cC^	81.92 %	3.45 %	74.16 %	87.74 %
TAP	Sows/gilts ^cB^	49.65 %	4.93 %	40.10 %	59.22 %
	Pre-weaning piglets ^aA^	28.59 %	4.55 %	20.55 %	38.27 %
	Nursery piglets ^bAB^	14.01 %	3.37 %	8.60 %	22.01 %
	Growing pigs ^cA^	26.83 %	4.41 %	19.08 %	36.32 %
	Finishing pigs ^dBC^	65.85 %	4.60 %	56.33 %	74.23 %
Total	Sows/gilts ^d^	36.61 %	2.75 %	31.40 %	42.15 %
	Pre-weaning piglets ^a^	27.43 %	2.58 %	22.66 %	32.78 %
	Nursery piglets ^b^	12.14 %	1.88 %	8.91 %	16.33 %
	Growing pigs ^a^	23.25 %	2.41 %	18.87 %	28.30 %
	Finishing pigs ^c^	74.28 %	2.50 %	69.08 %	78.88 %

Different lower case letters indicate the significant differences for categories in the same region and different capital letters indicate the significant differences for categories between regions. MBH-Metropolitan Region of Belo Horizonte; ZM-Zona da Mata; SSO-South/South West of Minas Gerais; TAP-Triangulo Mineiro/Alto Paranaíba; CI - confidence interval

### Risk factors associated with *L. intracellularis* infection

Four variables were considered associated to the *L. intracellularis* herd-level seropositivity (assistance at farrow and for the first colostrum suckle, cross fostering management, cleaning before disinfection and number of disinfectants used) in the primary univariable linear regression (*p*< 0.20) but only “cleaning before disinfection” showed a relationship to seropositivity (Table 3), with *p*< 0.05 in a multicolinear association.

**Table 3:** Linear regression results for risk factors. Only the “cleaning before disinfecting” showed a significant association to the L. intracelularis herd level seropositivity

Variable	*P* value	Regression coefficient		CI	
			Standard error	min	max
Newborn assistance at farrow and piglets’ first colostrum intake	0.22	0.17	0.14	−0.11	0.45
Cross fostering management	0.12	-.0.70	0.05	−0.17	0.03
Cleaning before disinfection	0.01	−0.04	0.02	−0.07	−0.01
Number of disinfectants used	0.11	0.01	0.04	−0.07	0.09

CI – Confidence interval

## Discussion

This is the first anti-*L. intracellularis* seroprevalence study using IPMA in intensive swine herds in Minas Gerais state, Brazil, one of the very few evaluations of seroprofiles of herds and risk factors for seropositivity. None of the included herds in the study were using the *L. intracellularis* attenuated vaccine. All of the herds had at least one seropositive sample, which demonstrates that *L. intracellularis* is endemic in the state, with an overall animal seroprevalence of 34.7 %. The relative low overall seroprevalence can be explained by the two potential seronegative categories, susceptible nursery piglets without seroconversion for *L. intracellularis* infection and multiparous sows that may previously have had IgG but not at the time of sampling, once the IgG titers decrease three months post infection [7].

Susceptibility to *L. intracellularis* infection starts in pigs of approximately 6 weeks of age and is followed by seroconversion 2 weeks later [13], which suggests increased IgG detection in animals older than 8 weeks. Therefore, detectable antibodies in grow/finishing pigs and sows/gilts likely arise from the immunological response due to direct exposition to the bacteria as none of the farms were current using a vaccine immunization protocol against *L. intracellularis*. These infection dynamics explain the higher seroprevalence for finishing pigs in all four regions. In addition, as nursery piglets younger than 5 weeks may have been sampled, the detected IgG antibodies in this phase may correspond to maternal antibodies; therefore, the positive results do not indicate an active immune response. To avoid this type of misinterpretation, only nursery pigs older than 5 weeks should be sampled.

The serological profiles obtained in the present study are consistent with the disease dynamics of infection that were previously observed in herds in which intensive antimicrobial protocols were in place for growing phases, without the restriction of growing promoters [7]. A total of 97 % of the sampled herds used antimicrobials as growth promoters and/or to prevent diseases, and all herds had at least one positive sample by IPMA, which indicated that drug usage does not prevent infection. In these production systems, usually, the infection tends to occur after the decrease of maternal antibodies (acquired via colostrum), a period that coincides with weaning stress, mixing pig groups (sometimes from different sources), as well as diet changes [7]. Antimicrobial usage reduces infection pressure in the nursery and, therefore, seroconversion begins in the growing strata, reaching peak levels at finishing phase and declining in sows that are kept in the herd for breeding [1, 7, 14]. In European herds, where the administration of antimicrobials as growth promoters is not allowed, seroconversion tends to occur earlier, about six weeks after weaning, corresponding to 70 days of age [15]. This probably reflects the nursery piglets susceptibility, in addition to the absence of antibiotics in feed, which make these pigs becoming infected and seroconverted earlier in relation to the herds there were sampled in the present study.

The sample weighting used in this study allowed an increase in the external validity and a better estimate of the prevalence of *L. intracellularis* infection in Minas Gerais and allowed comparisons to different sampled regions and pig categories. Without sample weighting, there may be a distortion of variance, with either overestimation or underestimation of the results [13]. Despite its importance, this type of analysis is not commonly used in other studies that compare crude seropositivity findings to either different categories or different country regions [16–18].

The only published study regarding *L. intracellularis* seroprevalence in Brazil, which used an indirect fluorescence antibodies method, demonstrated that 96.3 % of the herds from Minas Gerais were exposed to the bacteria [19], results very similar to our findings. Moreno et al. [9] sampled 19 herds in the same state, and 16 % had *L. intracellularis* DNA detected by PCR in fecal samples. Viott et al. [10] demonstrated the *L. intracellularis* presence in Minas Gerais herds, also using PCR, in co-infections in 47.8 % of the herds. These differences may be due to the low sensitivity of PCR, the small number of tested herds and samples or because PCR only detect shedders, whereas serology detects antibodies that last longer, suggesting a greater chance of detecting a seropositive animal, and consequently a positive herd [7]. Regardless of methodological differences, similar to our results, high herd prevalence was found in several countries worldwide, such as Canada [12], US [17], Australia [17], France, Spain [6], China [20], and Russia [21]. Despite the management and animal production law differences in these countries, *L. intracellularis* has been found in most herds.

Of more than 30 variables selected from the questionnaire, “cleaning before disinfecting” was indicated as a “protective factor”. Properties that adopted this management method reduced the seropositivity approximately 4 %, similar to the findings of Corzo et al. (2005) [12]. The correct management of facilities, with cleaning and disinfection, decreases environmental *L. intracellularis* maintenance [22, 23], which indicates in a lower infection pressure that prevents the infection to naïve pigs housed in the facilities.

The uniformity of sanitary management procedures among the studied herds may explain the lack of significant differences for both seropositivity and risk factors. This finding indicates that the included farms were homogeneous for management and biosecurity and were positive for *L. intracellularis* antibodies, the probability of finding a variable to discriminate positive and negative herds decreases as well as the differences in prevalence among regions. Other risk factors were previously found, but due the reasons mentioned above, they could not be associated to the infection in the present study. Solid and slatted floor in growing and finishing batches [24; 12] was adopted by 100 % of the properties research at our research. Herd size, pointed as a risk factor by Bronsvoort et al. (2001) [24] could not be statistically associated with the herd positivity, as the 30 properties were classified in the same strata [25]. The "all in-all out" system, indicated as a protective factor [16, 23] was adopted in 20 properties (66.7 %). Farrow-to-finish management and routinely use of antibiotics in feed were associated with susceptibility to contamination of Canadian herds [12], however, the surveyed herds showed a widespread practice (97 % of the properties). Cleaning and disinfection of the premises may also be related to lower prevalence [12], but 76.7 % of the 30 properties reported disinfect the facilities with some kind of disinfectant. Presence of water depth in the growing and finishing facilities, a potential characteristic for bacteria maintenance in the environment [22], was adopted by 90 % of the properties, a small discriminatory factor to associate with seropositivity.

## Conclusions

*L. intracellularis* antibodies were present in all of the sampled herds in Minas Gerais, which indicates the high circulation of the agent in the state and the overall weighted pig seroprevalence in Minas Gerais was 34.7 %. The predominant serological profile is consistent with that previously observed in herds from other countries, with finishing animals exhibiting the highest prevalence. “Cleaning before disinfecting” was a protective factor against *L. intracellularis* infection at the herd level.

## Methods

### Herd selection and sample collection

Samples for this cross-sectional study were obtained between May and August 2012, in farrow to finish commercial pig herds located in four major regions of intensive pig production of Minas Gerais: Triangulo Mineiro/Alto Paranaíba (TAP), Zona da Mata (ZM), Metropolitan Region of Belo Horizonte (MBH) and South/Southwest (SSO).

Herds and animal selection were performed by sampling in multiple stages [26]. Assuming a default value of 30 % seroprevalence, a 95 % confidence level, a 20 % absolute error and herd level population of 1400 (number of farms in the state of Minas Gerais), at least 21 herds were needed to estimate the seroprevalence [26]. Based on these criteria, 8 farms were sampled in the ZM region, 9 in SSO, 7 farms in MBH and 6 in TAP, for a total of 30 farms. Hog farm registration at the Instituto Mineiro de Agropecuária (IMA) until 2010 was adopted to select herds for convenience (primary units). The herd sensitivity, calculated for 20 % of herd prevalence was 98.1 %, using the HerdPlus routine (http://epitools.ausvet.com.au/). The property was considered positive if at least one pig was positive in the IPMA test.

Sampling within farms (secondary units) comprised 20 serum samples from animals of each category of production cycle (gilts and sows; pre-weaning piglets, 15 to 22 days of age; nursery, 22 to 60 days of age; growing pigs, 60 to 110 days of age; and finishing pigs, 110 to 160 days of age), considering 15 to 20 % prevalence of PPE and 95 % confidence interval [27]. The serological test used, imunoperoxidase monolayer assay (IPMA), has demonstrated to be 89 % sensitive and 100 % specific [28] . All bled animals were selected by random. Blood samples were collected by jugular vein puncture, identified, stored under refrigeration until natural coagulation/centrifugation and the serum samples stored at −20 °C until testing.

The Ethics Committee on Animal Use (CEUA) of the Universidade Federal de Minas Gerais approved this study.

### Questionnaire-based data collection

During each farm visit, a semi-structured questionnaire was used to collect relevant management and biosecurity information to characterize and identify risk factors related to herd-level *L. intracellularis* antibody positivity. The questions were about general herd management and biosecurity, facilities cleaning, antimicrobial use, quarantine, truck washer facilities, presence of water flow, piglet management, “all-in all-out” management, animal grouping, contact between animals from different batches, segregation of sick animals, and employee entrance protocols (Additional file1) . A formal consent for collecting samples and information through the semi-structured questionnaire was obtained from the farmers.

### Statistics

Data were entered into Excel (Microsoft Excel 2007) and analyzed using Stata, (version 12.0). The estimated prevalence, by region and strata, were calculated using the commands svyset < *animal identification variable* > [pweight = <*variable of weight*>], strata (<*region variable identification*>) to set the starting variables and svy command:prop < *variable with result* > for all data and strata (*if* option < *stratum variable* > = < *stratum code*>). These commands resulted in the weighted seroprevalence both for region and strata. Comparisons of strata and regions were performed using the adjusted Wald test, the test b command [p(line number) (column number)] = _b [p p (line number)(column number)].

Risk factors considered relevant to the enteric diseases were selected from the questionnaire and used to determine the association with seroprevalence at a herd level. Univariable linear regression was performed initially to predict the association of risk factors with the seroprevalence of *L. intracellularis* at the farm level, being selected those who had association with a liberal p-value of 0.20 or less (svy command: regress < answer variable > <independent variable>). After performing each of the univariable analyzes, we utilized a backwards method, manually, maintaining in the final model the variables with p < 0.05 and those that have been identified with multicolinear association with significant factors (variables) (Hosmer and Lemeshow, 2005).

## Additional file

Additional 1:
**Questionnaire survey used in the studied hog farms.** (PDF 218 kb)

## Abbreviations

IPMA: Immunoperoxidase Monolayer Assay
MBH: Metropolitan Region of Belo Horizonte
PPE: Porcine Proliferative Enteropathy
SSO: South/Southwest of Minas Gerais
TAP: Triangulo Mineiro/Alto Paranaíba
ZM: Zona da Mata
