# Supplementary material for: Serological profile, seroprevalence and risk factors related to Lawsonia intracellularis infection in swine herds from Minas Gerais State, Brazil
Source: BMC Vet Res. 2015 Dec 23;11:306. doi: 10.1186/s12917-015-0618-z (PMC4688965; doi:10.1186/s12917-015-0618-z)
Supplement: Additional 1: — Questionnaire survey used in the studied hog farms. (PDF 218 kb) [file 12917_2015_618_MOESM1_ESM.pdf]

Questionnaire Survey

1. Identification

Property's name:

Owner's name:

City:

Number of sows in the herd:
Breed:

2. Housing

( ) partially closed ( ) totally closed

3. Production system

( ) farrow to finish ( ) two sites (breeding herd-nursery and growth-finishing)
( ) three sites (breeding herd, nursery and growth-finishing)

4. Facilities

- Nursery

( ) allow contact between batches ( ) do not allow contact between batches

- Growing

( ) allow contact between batches ( ) do not allow contact between batches

- Finishing

( ) allow contact between batches ( ) do not allow contact between batches

5. Environment conditions

- Temperature

_ _ Nursery __ __Growing __ _Finishing

- Air flow

( ) natural ( ) mechanical Specify:

6. Herd practical procedures

- Colostrum and milk management

Newborn assistance at farrow and piglets' first colostrum intake?

( ) Yes ( ) No

Does the piglet suckle in the first hour after birth?

( ) Yes ( ) No

Is the litter managed in suckling groups?

( ) Yes ( ) No

Do you use sow milk replacer?

( ) Yes ( ) No

Do you keep a colostrum/milk storage?

( ) Yes ( ) No

- Weaning management

( ) classical - 21 days ( ) early segregated ( ) medicated early segregated

Piglet weaning age:

Cross-fostering:

( ) low transference rate ( ) high transference rate

( ) transference in the first 24 h after birth

( ) transference after 24 h after birth

- Nursery

Are pigs from different age mixed?

( ) Yes ( ) No

Are pigs from different sources mixed?

( ) Yes ( ) No

Do you have special management with wasted pigs?

What is batches size? What is the number of pigs/batches?

- Growing

Are pigs from different age mixed?

( ) Yes ( ) No

Are pigs from different sources mixed?

( ) Yes ( ) No

Do you have special management with wasted pigs?

What is batches size? What is the number of pigs/batche?

- Finishing

Are pigs from different age mixed?

( ) Yes ( ) No

Are pigs from different sources mixed?

( ) Yes ( ) No

Do you have special management with wasted pigs?

batches size: number of pigs/batche:

7. Nutrition

Do you use farm-made feed?

( ) Yes ( ) No

Do you use antimicrobials in the feed?

( ) Yes ( ) No

If yes, for which category are they provided?

- nucleus, microminerals, premix and vitamins

( ) farm-made ( ) acquired from nutrition companies

How is the feed provided?

What is the water source?

What is the drinker type?

How many drinkers/pigs?

8. Health management

What vaccines do you use and for what pig categories are they for?

What are the infectious agents diagnosed in the herd?

What actions do you have for their control?

Antimicrobials usage:

( ) to prevent ( ) to treat

What are the antimicrobials?

When are the pigs treated? (age)

Treatment length: Dosage:

9. Biosecurity

Pig flow in the property:

( ) continuous flow ( ) all-in, all-out

( ) farrowing batches

( ) nursery

( ) growing

( ) finishing

- Cleaning program

( ) daily cleaning ( ) cleaning after the pigs are moved

( ) Other

What detergents do you use:

How do you use them?

- Cleaning protocols

Do you do pre-clean? ( )Yes ( )No

What disinfectants do you use:

What is the final solution concentration?

What is the action time for the disinfectant?

What are other strategies do you use for cleaning or disinfection?

What is the carcass destination?

- People, vehicles and pigs entrance procedures

Vehicles

Do you have truck washer facilities? ( ) Yes ( ) No

Are the trucks disinfected before the entrance in the farm? ( ) Yes ( ) No

Are their cleaning and disinfection part of the routine? ( ) Yes ( ) No

People

Are the employees required to take a shower before entrance? ( ) Yes ( ) No

What kind of clothes does the employee wear during the working time?
( ) property uniform ( ) regular clothes

Pigs

How do you replace your breeding pigs?

( ) auto-replacement ( ) acquired replacement animals from multipliers

How often do you acquire animals from other sources?

Do you have quarantine? ( ) Yes ( ) No

How do you manage the pigs there?

Are these pigs kept in isolation?
For how long?
